# Inquiry on the Cure of Bronchocele, &c.

**Published:** 1812-02

**Authors:** 


*To the Editors of the Medical and Physical Journal.*

GENTLEMEN,

I LATELY read an account of a Cure for *Bronchocele*, by some *external* means, and effected in a very short time; whether with the assistance of emetics or not, I do not recollect.

I also lately read an explanation of the cause of *short sight*, different from that we have been long accustomed to give, viz. the too convex form of the cornea, and which, I think, attributed that kind of vision to the action of the muscles alone.*

Will you give me leave to inquire, through the medium of your Journal, whether the above subjects have been lately mentioned; or if you, or any of your readers, can give the history of the means of cure in Bronchocele I have alluded to, or the new opinion as to the kind of vision I have mentioned, it will oblige, Gentlemen,

*Dec.* 11, 1811. An OLD CORRESPONDENT.

The mode of cure in Bronchocele by means of *burnt sponge*, &c. as proposed by Prosser, is often efficacious, but it is very tedious, and also expensive.

---

* In the Phil. Trans. Part II. p. 378, for the year 1811, our correspondent will find a detailed statement of the facts respecting a peculiarity of the eye, to which he alludes, in a Paper by Dr. W. C. Wells, with the title of "Observations and Experiments on Vision." In a former number of our Journal, in the autumn of 1811, we presented our subscribers with a short notice of this paper, then in the course of being read before the Royal Society; and from that, probably, our correspondent had the first intimation of Dr. Wells's Experiments and Opinions upon a State of the Organ of Vision, so different from what has been generally apprehended. From the great price that must be paid for the few articles strictly connected with the medical profession, which appear in the Phil. Trans. as well as from the general merit of those articles, we have been accustomed to present our readers with copious transcripts from them: and the paper by Dr. Wells, which we think highly ingenious, will afford excerpts for our next number.—EDITORS.

COLLECTANEA

